# Fat-enlarged axillary lymph nodes are associated with node-positive breast cancer in obese patients

**DOI:** 10.1007/s10549-021-06262-z

**Published:** 2021-06-03

**Authors:** Roberta M. diFlorio-Alexander, Qingyuan Song, Dennis Dwan, Judith A. Austin-Strohbehn, Kristen E. Muller, William B. Kinlaw, Todd A. MacKenzie, Margaret R. Karagas, Saeed Hassanpour

**Affiliations:** 1 Department of Radiology, Dartmouth-Hitchcock Medical Center, 1 Medical Center Drive, Lebanon, NH 03756 USA; 2 Department of Biomedical Data Science, Dartmouth College, 1 Medical Center Drive, HB 7261, Lebanon, NH 03756 USA; 3 Department of Internal Medicine, Carney Hospital, 2100 Dorchester Ave, Dorchester, MA 02124 USA; 4 Department of Pathology, Dartmouth-Hitchcock Medical Center, 1 Medical Center Drive, Lebanon, NH 03756 USA; 5 Department of Medicine, Dartmouth-Hitchcock Medical Center, 1 Medical Center Drive, Lebanon, NH 03756 USA; 6 Department of Epidemiology, Dartmouth College, 1 Medical Center Drive, Lebanon, NH 03756 USA; 7 Department of Computer Science, Dartmouth College, Hanover, NH 03755 USA

**Keywords:** Axillary lymph node, Metastasis, Breast cancer, Obesity, Breast MRI, Mammography

## Abstract

**Purpose:**

Obesity associated fat infiltration of organ systems is accompanied by organ dysfunction and poor cancer outcomes. Obese women demonstrate variable degrees of fat infiltration of axillary lymph nodes (LNs), and they are at increased risk for node-positive breast cancer. However, the relationship between enlarged axillary nodes and axillary metastases has not been investigated. The purpose of this study is to evaluate the association between axillary metastases and fat-enlarged axillary nodes visualized on mammograms and breast MRI in obese women with a diagnosis of invasive breast cancer.

**Methods:**

This retrospective case–control study included 431 patients with histologically confirmed invasive breast cancer. The primary analysis of this study included 306 patients with pre-treatment and pre-operative breast MRI and body mass index (BMI) > 30 (201 node-positive cases and 105 randomly selected node-negative controls) diagnosed with invasive breast cancer between April 1, 2011, and March 1, 2020. The largest visible LN was measured in the axilla contralateral to the known breast cancer on breast MRI. Multivariate logistic regression models were used to assess the association between node-positive status and LN size adjusting for age, BMI, tumor size, tumor grade, tumor subtype, and lymphovascular invasion.

**Results:**

A strong likelihood of node-positive breast cancer was observed among obese women with fat-expanded lymph nodes (adjusted OR for the 4th vs. 1st quartile for contralateral LN size on MRI: 9.70; 95% CI 4.26, 23.50; *p* < 0.001). The receiver operating characteristic curve for size of fat-enlarged nodes in the contralateral axilla identified on breast MRI had an area under the curve of 0.72 for predicting axillary metastasis, and this increased to 0.77 when combined with patient and tumor characteristics.

**Conclusion:**

Fat expansion of axillary lymph nodes was associated with a high likelihood of axillary metastases in obese women with invasive breast cancer independent of BMI and tumor characteristics.

**Supplementary Information:**

The online version contains supplementary material available at 10.1007/s10549-021-06262-z.

## Introduction

Obesity affects more than 30% of adult women worldwide, and obese women have an increased risk of breast cancer with increased risk of axillary node metastases and breast cancer-specific mortality compared to normal-weight women [1, 2]. Decreased survival is experienced by both pre-menopausal and post-menopausal obese women. Studies have reported that there are no appreciable differences in mammography screening rates and screening intervals among obese women and normal-weight women suggesting that biologic factors are likely responsible for higher breast cancer risk and mortality [2–5]. While the exact mechanisms accounting for the poor prognosis of breast cancer in obese women are not fully understood, increased available estrogen, insulin, and tumor-promoting characteristics of dysregulated obese adipose tissue have been proposed as contributing factors [1, 6, 7]. Axillary lymph node (LN) status is one of the most important independent prognostic indicators of survival, with reports of up to 14% decrease in 5-year survival associated with a single metastatic axillary node [8, 9]. These findings suggest that targeting nodal metastases in breast cancer treatment may significantly impact breast cancer mortality.

**Fig. 1:**
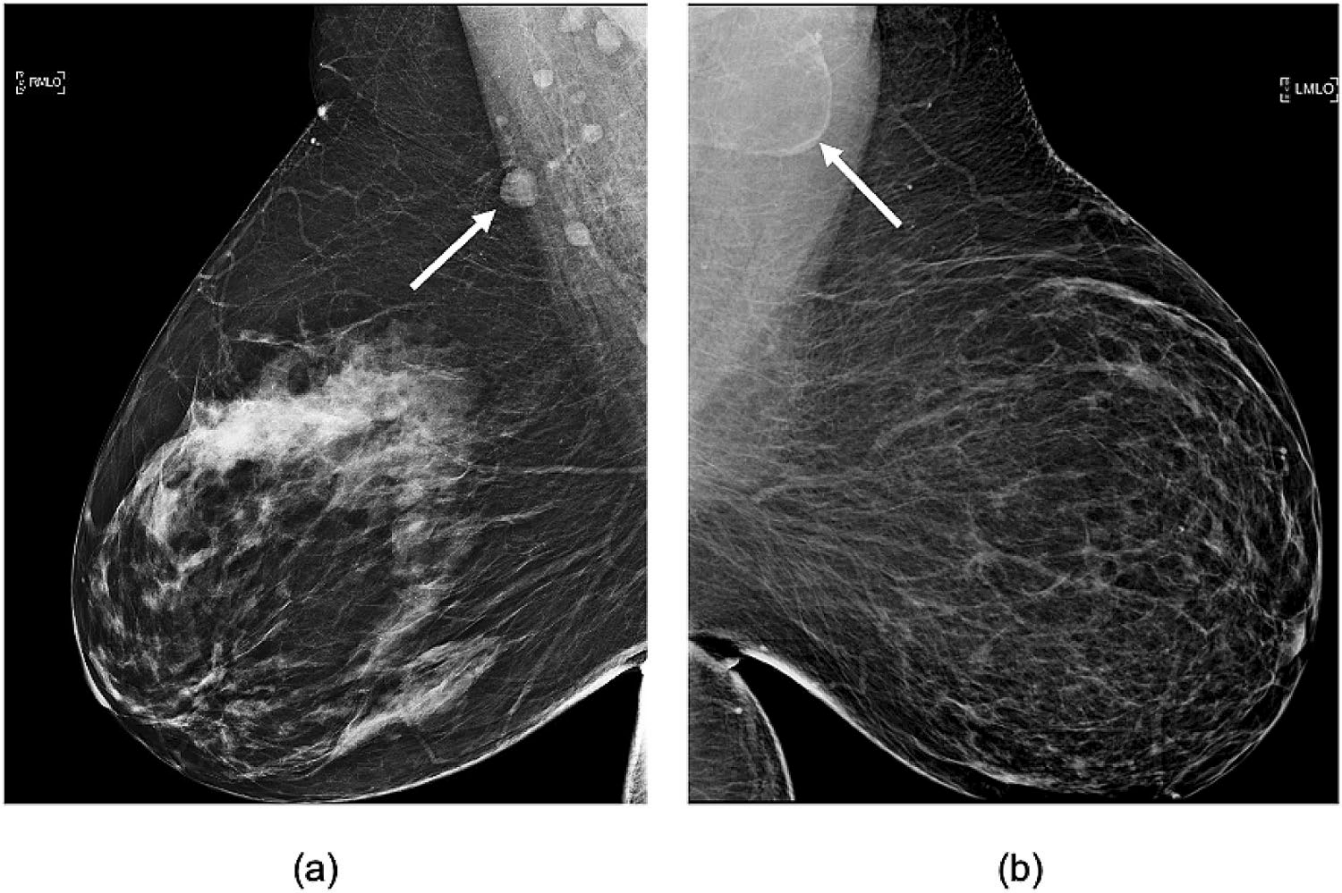
Variable axillary lymph node size and morphology on mammography. Obese women with variable fatty node morphology in the axilla on mediolateral oblique (MLO) views. **a** Normal axillary LNs measuring less than 1.5 cm in a 63 y/o woman with BMI = 43.2. **b** fat-infiltrated LN measuring 4.2 cm in a 52 y/o woman with BMI = 45.8. Arrows point to the largest visible axillary lymph node

Recent studies have found that obesity is associated with enlarged axillary LNs on screening mammograms secondary to fat expansion of the radiolucent LN hilum without accompanying enlargement of the nodal cortex as demonstrated in Fig. 1b [10, 11]. There is marked variability in the degree of fatty node enlargement among obese women with similar body mass index (BMI), yet the clinical significance of variable benign LN size and morphology is unknown (Fig. 1). The morphology of enlarged fat-expanded lymph nodes is distinctly different from nodal enlargement due to reactive or malignant adenopathy. Fatty nodes reflect fat deposition within the central hilum and are often associated with a thin, effaced peripheral cortex rather than a thickened or enlarged cortex that is characteristic of reactive hyperplasia or axillary metastases. Obesity-related fat deposition in other organs such as the liver, kidney, and bone marrow is associated with altered lipid metabolism that may lead to organ dysfunction, increased risk of malignancy, and poor cancer outcomes [12–16]. There are several proposed mechanisms for obesity-associated poor-prognosis cancer. Obesity-related adipose inflammation and dysregulated lipid metabolism can lead to increased secretion of inflammatory markers and adipokines that promote angiogenesis and tumor growth. Surplus local fat may be used as fuel by malignant cells and may additionally provide essential phospholipid building blocks required for cell membrane synthesis within proliferating tumors [17]. However, there has been very little research exploring fat deposition within LNs, the organelles of the immune and lymphatic system distributed throughout the body. We hypothesized that fat-infiltrated axillary LNs may be subject to similar adipose-induced dysfunction identified in other organs infiltrated by fat; and that the changes exerted by excess hilar fat may impact host resistance, potentially contributing to a higher risk of axillary metastases. Studies evaluating the significance of fat-expanded nodes may improve our understanding of mechanisms responsible for increased risk of node-positive, poor-prognosis breast cancer among obese women. Our study aimed to evaluate the relationship between axillary metastases and the size of fatty axillary nodes visualized on breast MRI and mammograms among obese women with invasive breast cancer.

## Materials and methods

### Data collection

This retrospective case–control study was approved by an institutional review board and was compliant with the Health Insurance Portability and Accountability Act. The dataset was collected from the Institutional Cancer Center Database identifying all obese women (BMI > 30) with histologically confirmed invasive breast cancer diagnosed between April 1, 2011 and March 1, 2020. An institutional review board exempted informed consent from these patients. Exclusion criteria included: imaging studies not available for review, pathology report not available for review, age greater than 89, history of recurrence, adenopathy secondary to malignancy other than breast cancer, isolated tumor cells on node histology, status of lymphovascular invasion (LVI) not available, or bilateral synchronous breast cancer with bilateral axillary metastases. Patients’ nodal status was determined according to their surgical pathology report or pre-operative LN biopsy histology report in patients who had neoadjuvant chemotherapy prior to surgery. All histologically confirmed node-positive patients that fulfilled the inclusion criteria were included in the final dataset. Node-negative patients were randomly selected from the same time period and were subjected to the same inclusion and exclusion criteria, in a ratio of approximately one control to two cases.

We collected the following patient and tumor characteristics from the electronic medical record as potential confounders: patient’s age at initial diagnosis, BMI at the time of diagnosis, tumor size, tumor grade, estrogen receptor (ER), progesterone receptor (PR), and human epidermal growth factor receptor 2 (HER2) status, presence of LVI, and treatment with neoadjuvant chemotherapy or neoadjuvant endocrine therapy. For patients treated with neoadjuvant systemic therapy, tumor size on pre-treatment breast MRI was used in the analysis, while tumor size from surgical pathology reports was used for all other patients.

### Image analysis

**Fig. 2:**
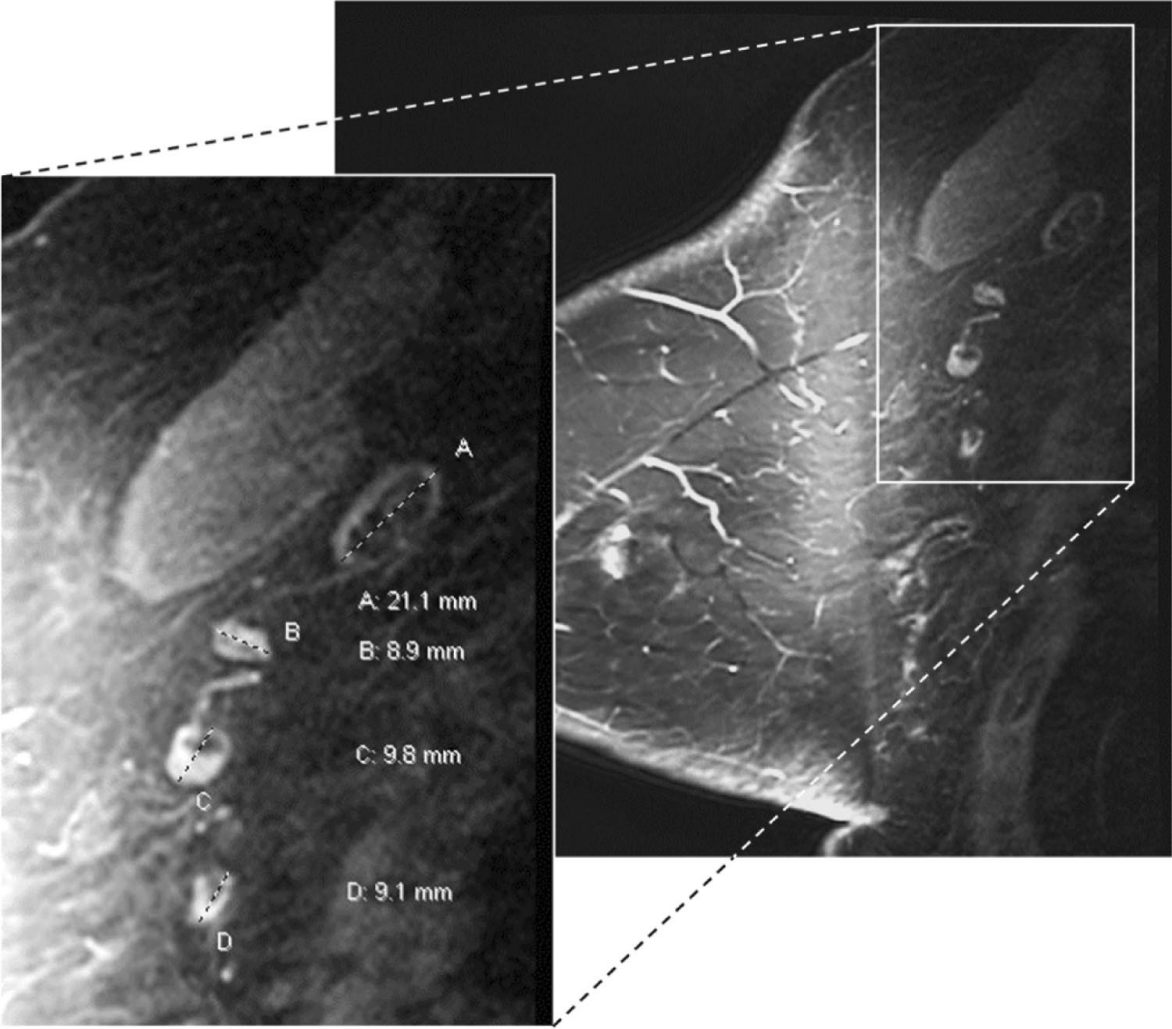
Variable axillary lymph node size and morphology on breast MRI. 65 y/o female with ER+ HER2− infiltrating ductal cancer of the left breast showing benign variable lymph node size and morphology in the right axilla contralateral to the known breast cancer. Contrast-enhanced fat-saturated sagittal MRI image of the right breast, and enlarged view of the right axilla demonstrate variable nodal size and morphology. Fat-enlarged node with expanded fatty hilum measures 21 mm in length (A) while normal nodes with small fatty hila measure less than 10 mm (B, C, D). The largest visible axillary node of 21 mm was chosen as the index node for the analysis in our study

**Fig. 3:**
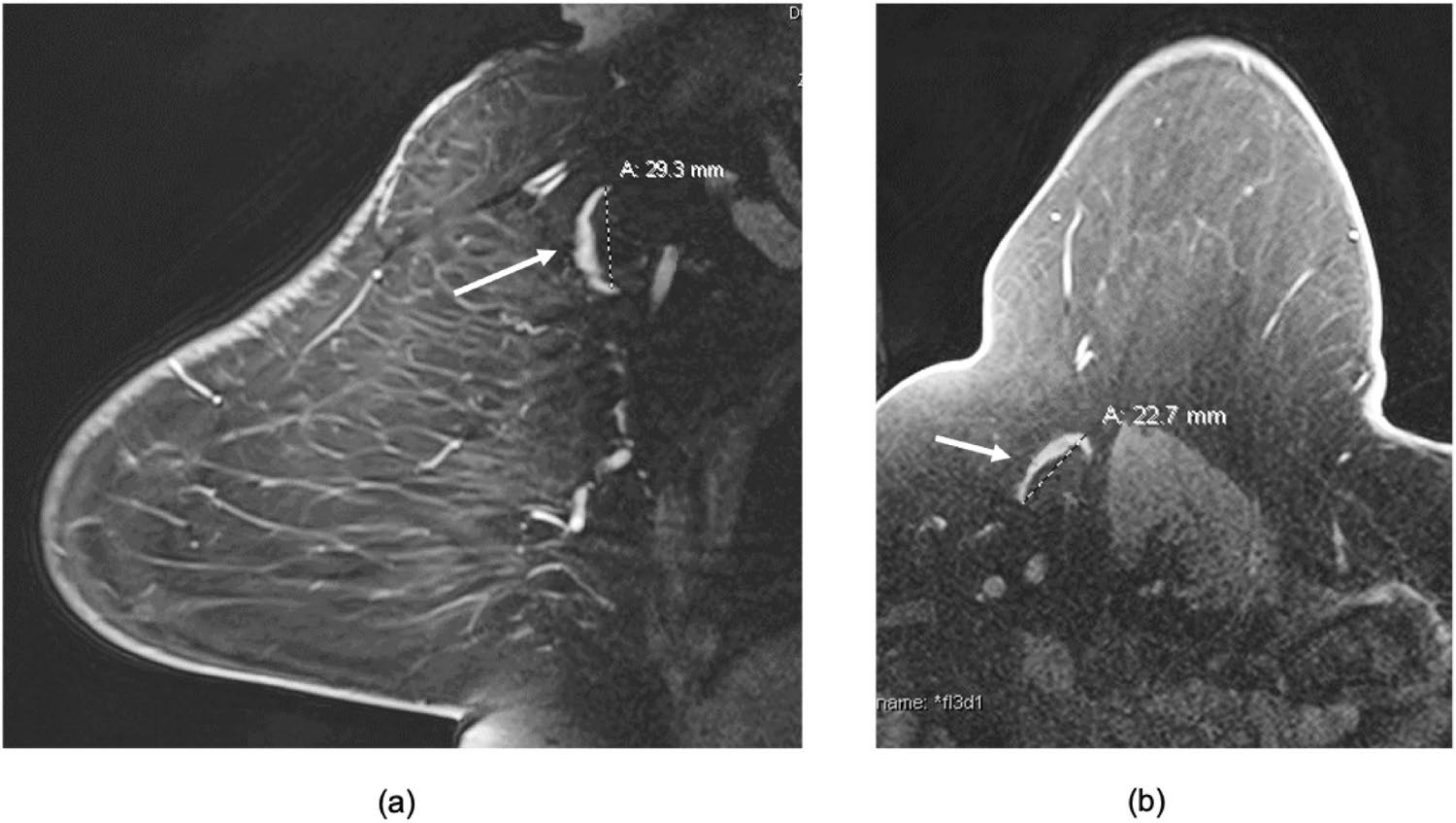
Axillary node measurements on breast MRI. 71 y/o female with left breast 18 mm ER+ HER2− node-positive invasive ductal cancer showing differences in the measurements of a single lymph node in the contralateral right axilla in the sagittal and axial plane. **a** Sagittal contrast enhanced fat-saturated breast MRI image through the contralateral right axilla demonstrates a fat-enlarged lymph node measuring 29 mm in greatest dimension. **b** Axial contrast enhanced image through the same index node shows smaller greatest dimension of 23 mm. We chose the largest measurement (in the sagittal or axial plane) for our study and in this case, we used 29 mm for the analysis as identified in the sagittal plane from (**a**)

Axillary LNs were measured on pre-treatment and pre-operative breast MRI and mammograms by a breast radiologist with 18 years of experience. To assess the inter-observer agreement of LN measurements, a second breast radiologist with 19 years of experience independently measured LN size for 28% of the patients randomly selected from the original dataset. We used the largest LN visualized on breast MRI in the contralateral axilla for our primary analysis. The single largest LN within the axilla was chosen as the index node and measured along its greatest longitudinal axis in the axial or sagittal plane as shown in Figs. 2 and 3. Analysis of lymph node measurements visualized mammographically in the contralateral axilla is available in the Supplementary Material and measured as described in the prior study [10]. In order to avoid potential inclusion of morphologically normal nodes with micro-metastases occult to imaging in the ipsilateral axilla, we did not include measurements of ipsilateral axillary lymph nodes on MRI or mammography in the primary analysis, but they are available in the Supplementary Materials. Images were reviewed on Barco 3-megapixel MDCG-3221 monitors (Kortrijk, Belgium) with Philips PACS v. 3.6 (Philips Healthcare; Best, Netherlands).

### Statistical analysis

We conducted an independent sample t-test to compare the mean index LN size of the largest visible axillary node on breast MRI and mammograms. We further calculated the pairwise Pearson correlation between the index LN sizes measured on breast MRI and mammograms. Pearson correlation also was used to evaluate the inter-observer reliability of LN measurements between two radiologists. We examined the association between node-positive breast cancer and fat-enlarged axillary LN size using multivariate logistic regression to adjust for covariates of interest. We conducted separate analyses on LNs visualized on breast MRI and mammograms. In each analysis, LN measurements were categorized into quartiles containing equal numbers of cases. The quartile with the smallest LN sizes was used as the reference group in the regression analyses. Adjusted odds ratios (OR) with 95% confidence intervals (CI) were calculated for LN size quartiles. All statistical analyses were performed using R software (version 4.0.3; RStudio, Boston, Mass). Receiver operative characteristic (ROC) curves with fivefold cross-validation were generated to evaluate the ability to discriminate node-positive from node-negative patients using LN size. We illustrated ROC curves of three logistic regression models: first, using LN size alone as the predictor; second, using the collected clinical variables including age, BMI, tumor size, tumor grade, and LVI; and lastly using a combination of LN size and collected variables. ROC curves and cross-validation were done with Python programming language (Version 3.7.1).

## Results

**Fig. 4:**
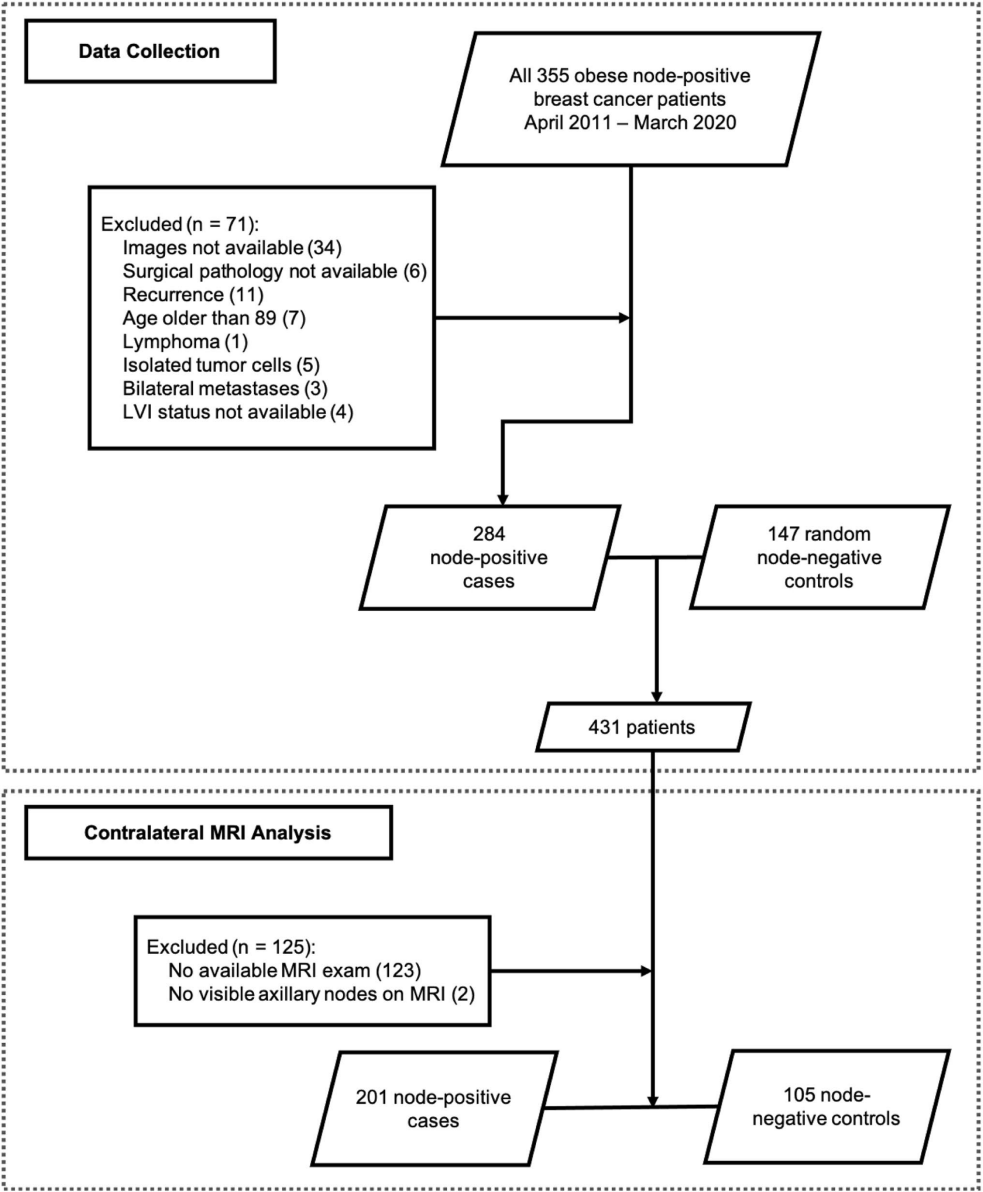
Flowchart of data collection for primary analysis evaluating LN size on breast MRI identified in the axilla contralateral to the known breast cancer

A total of 355 patients with node-positive breast cancer and BMI > 30, diagnosed between April 1, 2011, and March 1, 2020, were identified by the Institutional Cancer Center Database. 71 patients were excluded, and the reasons are shown in Fig. 4. The remaining 284 node-positive cases were combined with 147 random-selected node-negative controls to form our final dataset of 431 patients.

**Fig. 5:**
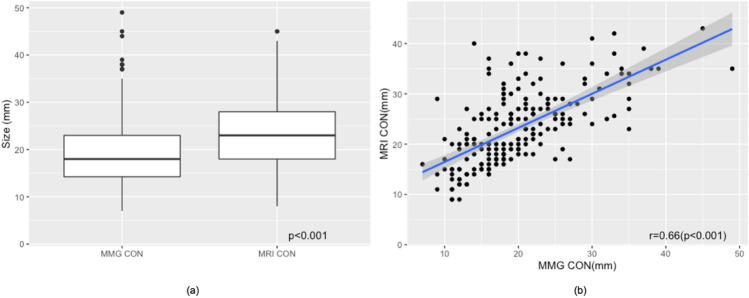
Correlation of LN measurements on mammogram and breast MRI. **a** Distribution of axillary LN sizes measured on contralateral mammograms and breast MRI. **b** Despite difference in mean size, LN measurements obtained mammographically and on breast MRI were positively correlated (*r* = 0.66, *p* < 0.001). Scatterplot of LN size on mammogram and breast MRI with fitted regression line (blue) and 95% CI (shaded) showing good correlation. *MMG *mammogram, *CON *contralateral

**Fig. 6:**
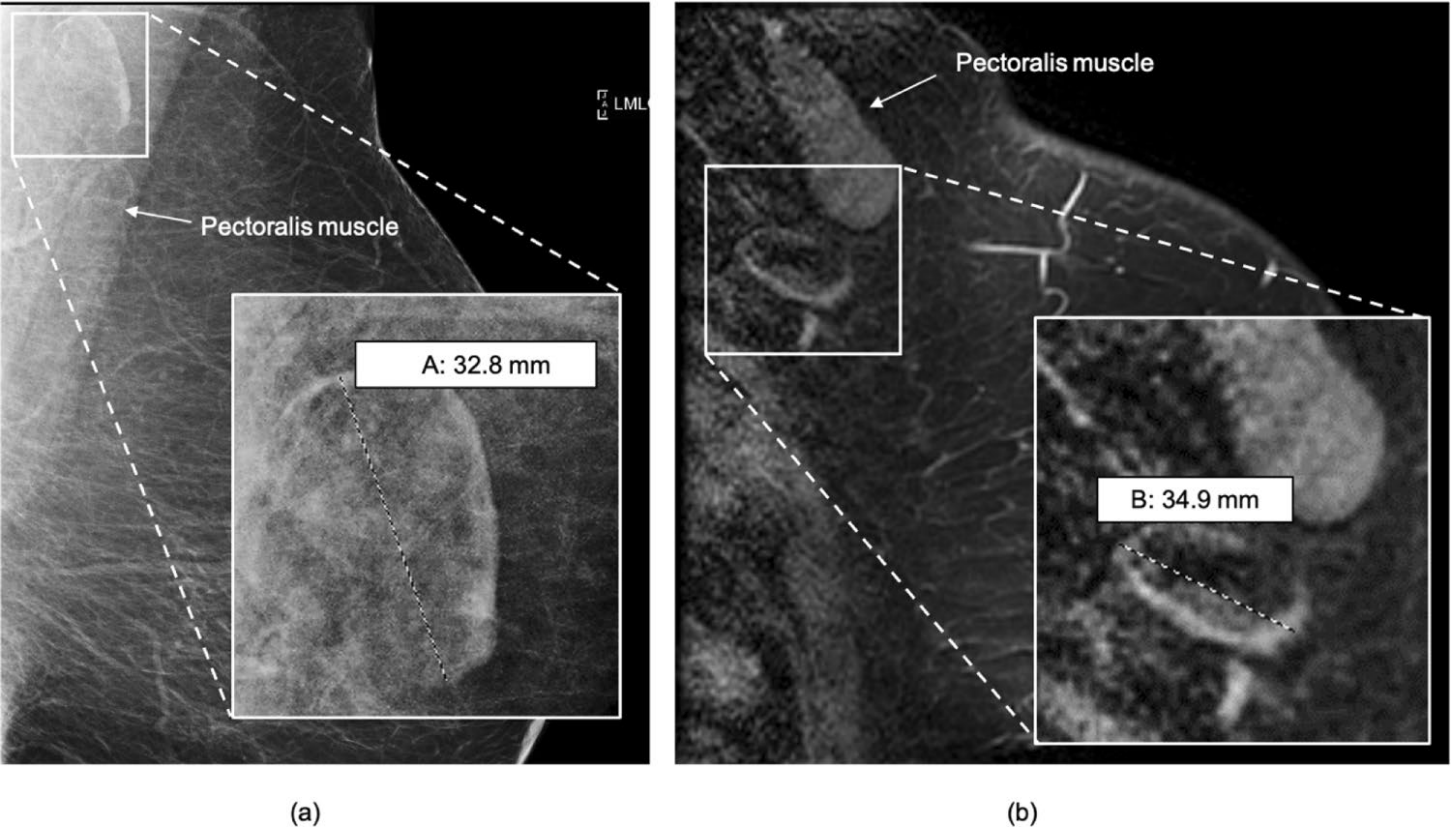
Comparison of LN measurements on mammogram and breast MRI. 54-year-old female with right breast ER+ HER2+ IDC demonstrating contralateral fat-expanded node on mammography compared to breast MRI. **a** Left MLO digital mammogram shows a fat-expanded lymph node in the contralateral axilla measuring 33 mm in largest dimension, **b** sagittal fat-saturated enhanced breast MRI of the same patient demonstrates a slightly larger size of the same lymph node measuring 35 mm in length on MRI

We observed that LN sizes visualized on mammography were significantly smaller than those identified on breast MRI with mean size of 19.70 ± 7.42 mm on mammography, and 25.54 ± 7.29 on breast MRI (*p* < 0.001). Despite difference in mean size, LN measurements obtained mammographically and on breast MRI were positively correlated (*r* = 0.66, *p* < 0.001) (Figs. 5, 6).

LNs were almost always identified on breast MRI consistent with a larger field of view that usually allows comprehensive evaluation of the entire axilla and demonstrates a larger number of axillary nodes. Based on the second radiologist’s assessment of 28% of patients, the measurement of contralateral LNs on MRI showed strong inter-observer agreement with a Pearson correlation coefficient of 0.67 (*p* < 0.001). Our primary analysis consists of LN measurements in the contralateral axilla on breast MRI. The following patients were excluded: 123 patients without a breast MRI available for review, and 2 patients without visible contralateral axillary lymph nodes on breast MRI. We observed that patients with breast MRI were younger (mean age of 58.68 years versus 66.34, *p *value < 0.001), had slightly lower BMI (mean 35.99 versus 37.60, *p *value = 0.02), and had a larger tumor (mean size 33.46 mm versus 27.54 mm, *p *value = 0.013). No significant difference was found for tumor grade, molecular subtype, and nodal status between patients who did and did not have a breast MRI. As a result, a total of 306 patients (201 cases and 105 controls) were included in the primary analysis of MRI-visualized contralateral axillary lymph nodes (Fig. 4). Contralateral LN sizes on breast MRI were significantly correlated with BMI (Pearson correlation coefficient: 0.14, *p *value = 0.015). The demographics and clinical characteristics of the patients in the primary analysis of MRI-visualized contralateral axillary lymph nodes are shown in Table 1. The patient demographics and analysis results of contralateral mammographic LNs and of ipsilateral MRI and mammographic LNs can be found in the Supplementary Materials.

**Table 1:** Characteristics of patients used for contralateral MRI analysis (*n* = 306)

	Node negative	Node positive	*p *value
*N* (%)	105 (34.3)	201 (65.7)	
Age (years, SD)	60.67 (10.1)	57.73 (10.6)	0.020
BMI (SD)	36.33 (5.8)	35.86 (4.9)	0.458
Tumor size (mm, SD)	25.83 (17.1)	37.52 (23.8)	< 0.001
Tumor grade (%)			0.032
1	23 (21.9)	22 (10.9)	
2	47 (44.8)	96 (47.8)	
3	35 (33.3)	83 (41.3)	
Molecular subtypes (%)			0.022
ER + HER2-	81 (77.1)	147 (73.1)	
HER2+	9 (8.6)	38 (18.9)	
TNBC	15 (14.3)	16 (8.0)	
NAC or NAE (%)	5 (4.8)	56 (27.9)	< 0.001
LVI (%)	22 (21.0)	117 (58.2)	< 0.001
MRI CON LN size (mm, SD)	19.91 (6.93)	25.43 (7.0)	< 0.001
[8,18] (%)	54 (51.4)	31 (15.4)	
(18, 23] (%)	23 (21.9)	49 (24.4)	
(23, 28] (%)	15 (14.2)	59 (29.4)	
(28, 45] (%)	13 (12.4)	62 (30.9)	

*BMI* body mass index, *ER* estrogen receptor, *HER2* human epidermal growth factor receptor 2, *TNBC* triple negative breast cancer, *NAC* neoadjuvant chemotherapy, *NAE* neoadjuvant endocrine, *LVI* lymphovascular invasion, *CON* contralateral, *LN* lymph node

### Association between fatty nodes and nodal status

Compared to the reference quartile (LN < 18 mm), statistically significant positive associations were observed between node-positive breast cancer and larger fatty nodes in the contralateral axilla on breast MRI adjusting for age, BMI, tumor size, tumor grade, tumor molecular subtype, and LVI. Specifically, contralateral LN size greater than 28 mm (4th quartile) had an estimated odds ratio of 9.70 compared to the first quartile (95% CI 4.26, 23.50; *p *value < 0.001), and the estimated odds ratio increased with nodal size (Table 2). We observed a similar positive association between axillary LN size and node-positive breast cancer with contralateral mammographic LN, and ipsilateral mammographic and breast MRI-visualized LNs as shown in Supplementary tables S2 and S3. Of note, contralateral LN size significantly correlated with ipsilateral LN size, assuring the validity of using contralateral LNs for the primary analysis (Supplementary Materials Figure S1).

**Table 2:** Multivariate logistic regression analysis of association between index LN size on contralateral MRI and axillary metastases adjusting for potential confounders (*N* = 306)

Variables	201 cases, 105 controls		
	OR	95% CI	*p *value
MRI CON LN (mm)			
[8,18]			Reference
(18, 23]	4.14	(1.97, 8.96)	< 0.001
(23, 28]	6.37	(2.87, 14.81)	< 0.001
(28, 45]	9.70	(4.26, 23.50)	< 0.001
Age	0.97	(0.94, 1.00)	0.061
BMI	0.95	(0.90, 1.00)	0.072
Tumor size	1.02	(1.01, 1.04)	0.008
Tumor grade			
1			Reference
2	1.67	(0.74, 3.77)	0.218
3	2.52	(1.00, 6.45)	0.050
Subtypes			
ER+			Reference
HER2+	1.35	(0.51, 3.05)	0.528
TNBC	0.26	(0.08, 0.88)	0.017
LVI	3.84	(2.10, 7.24)	< 0.001

### Association between fatty LN size and nodal status in patients without LVI

In multivariate regression, LVI remained strongly associated with positive nodal status, and we observed larger LN sizes in patients with LVI compared to those without (*p* < 0.001). From our dataset, 167 patients had no LVI, of whom 84 (50.3%) had nodal metastasis. We therefore performed additional analysis to test the association between fat-expanded nodes and axillary metastases in patients without LVI. Our analysis showed that for patients without LVI, increased LN size was associated with an increased likelihood of nodal metastasis, adjusting for other collected variables. The association between contralateral LN size and nodal metastasis in patients without LVI is shown in Table 3.

**Table 3:** Association between LN size and axillary metastases in patients without LVI

MRI CON LN (mm)	No LVI (84 cases, 83 controls)		
	OR	95% CI	*p *value
[8,17]			Reference
(17, 21]	5.26	(1.83, 16.28)	< 0.003
(21, 27]	9.23	(3.32, 28.22)	< 0.001
(27, 40]	17.46	(5.76, 60.20)	< 0.001

The analyses were adjusted for potential confounders including age

*BMI* tumor size and grade, molecular subtypes, and the presence of LVI (results not shown in the table), *CON* contralateral

### Predictability of axillary node metastases by size of fat-enlarged contralateral axillary LNs

**Fig. 7:**
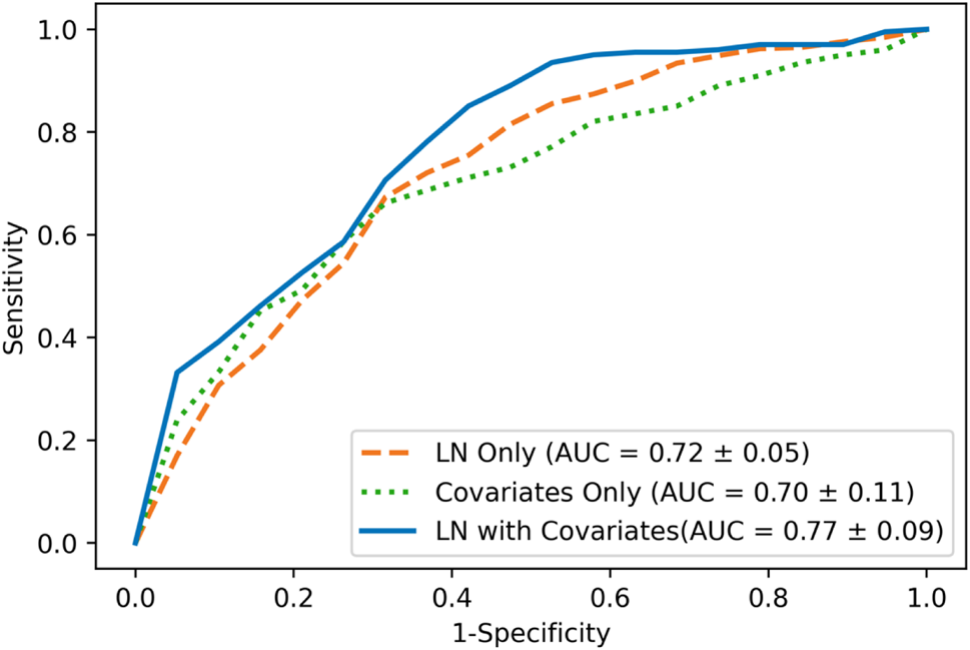
Performance of contralateral fat-enlarged node size for predicting axillary metastases. Mean ROC curves of node positive breast cancer prediction using axillary LN size from contralateral MRI with fivefold cross-validation. The orange dashed line indicates prediction of axillary metastases using contralateral MRI LN size alone. The green dotted line indicates the predictions using collected variables including patients’ age and BMI at diagnosis, tumor size, tumor grade, molecular subtype, and LVI. The blue solid line indicates the predictions of axillary metastases using contralateral LN size combined with other variables

Figure 7 shows the ROC curves for axillary metastasis using LN size alone and combining LN size with patient and tumor characteristics, including age, BMI, tumor size, tumor grade, molecular subtype, and LVI. The logistic regression model using the index LN size from contralateral MRI achieved an area under the ROC curve (AUC) of 0.72 ± 0.05. ROC curve using only combined patients and tumor characteristics resulted in a slightly lower AUC of 0.70 ± 0.11, indicating that LN size had a better ability to discriminate nodal status than clinical variables alone. ROC curve using a combination of LN size with clinical variables together improved the AUC to 0.77 (Fig. 7). The result of model’s predictability was consistent for LN size from ipsilateral breast MRI, and bilateral axillary LNs on mammograms with the corresponding ROC curves shown in Supplementary Materials S3.

## Discussion

We found that enlarged fat expanded axillary lymph nodes were strongly associated with node-positive breast cancer in obese women despite age, BMI, and tumor characteristics. Previous studies have shown that obesity is associated with an increased size of axillary LNs due to hilar fat expansion without associated increase in cortical size, a finding demonstrated by increased hilo-cortical ratio in enlarged nodes of obese women [10]. This morphology is in contrast to the diffuse cortical thickening seen with reactive adenopathy. While prior studies showed that hilar fat expansion accounted for nodal enlargement of fatty nodes, overall LN length was the most sensitive metric, and therefore was used to measure the degree of LN fat expansion in our study. With the potential use of fat-enlarged LN as a clinically useful marker in the future, larger studies could identify and evaluate the predictive value of additional LN characteristics.

The area under the ROC curve for the association between axillary metastases and enlarged fatty nodes in the contralateral axilla on breast MRI was 72%, and this increased to 77% when combined with other clinical variables. Breast MRI affords visualization of a larger number of axillary LNs compared to mammography due to a larger field of view that includes most level 1, 2, and 3 axillary nodes compared to partial visualization of level 1 axillary nodes on mammograms [9, 18]. The mean size of MRI-detected nodes was significantly larger than mammographically detected nodes, likely reflecting improved visualization of the axilla, and possible differences in LN size related to patient position, degree of compression, multiple imaging planes, and improved resolution of nodes on MRI compared to mammography. Increased mean size of axillary nodes on MRI was unlikely to be caused by the growth of the index LN between the mammogram and MRI that was obtained on average 2 weeks after the breast cancer diagnosis. Contralateral nodes identified by MRI were chosen for the primary analysis in our study to avoid the potential inclusion of morphologically normal nodes with micro-metastases occult to imaging in the ipsilateral axilla, and to permit the largest number of visible axillary nodes for analysis. Interestingly, both ipsilateral and contralateral enlarged fatty lymph nodes visualized on breast MRI and mammography were independently associated with nodal metastases as indicated in the supplementary material. Future studies should aim to compare the predictive nature of fatty nodes on different imaging modalities and to identify which modality provides the most clinically useful tool.

Ectopic fat deposition within organs has been shown to represent a better predictor of adverse health outcomes, increased malignancy risk, and poor cancer outcomes compared to BMI or subcutaneous fat [14, 19–23]. The growing field of body composition research and the evaluation of fat distribution has improved our understanding of poor cancer outcomes among the obese population [21]. Our study demonstrated that fatty nodes were strongly associated with axillary metastases in obese patients, while BMI was not. While there is a lack of research evaluating LN fat deposition in humans, findings in obese mice have demonstrated impaired immune function and decreased lymphatic transport linked to adipose accumulation within LNs and lymphatics [24]. Within the breast, studies evaluating lipid function and metabolism have found associations between altered fat composition and breast cancer. Changes in fatty acid oxidation are associated with increased breast cancer proliferation and poor outcomes [17, 25]. High expression of Spot14, a requisite gene for fatty acid synthesis, is associated with decreased survival in breast cancer patients [26]. Differences in fatty acid fractions have been observed in breast adipose tissue of postmenopausal women with breast cancer compared to women without breast cancer, independent of BMI [27]. Additionally, MR spectroscopy has identified lipid dysregulation within breast tissue of women with BRCA gene mutations [27].

LVI indicates the presence of tumor cells within the peri-tumoral vascular or lymphatic channels and is a strong prognostic marker for axillary metastases. LVI is therefore included as a predictive feature in models designed to assess the likelihood of positive sentinel nodes as well as positive non-sentinel node axillary metastases [28, 29]. In our study, half of the patients without LVI developed nodal metastasis prompting us to perform additional analysis to better understand the relationship between fat-enlarged nodes and nodal metastasis in patients without LVI. Our findings demonstrated a strong association between fatty node size and axillary metastasis among patients without LVI. A proposed mechanism for this interesting observation may be that LVI and fat-enlarged nodes operate via divergent mechanisms in the invasion-metastasis cascade. The status of the axilla in breast cancer patients reflects the interaction between tumor aggressiveness and host resistance. LVI is most commonly seen with larger tumor size and higher histologic grade, suggesting that aggressive tumor characteristics influence the predictive nature of LVI [30]. In contrast, the association between fat-enlarged nodes and axillary metastases in women without LVI may reflect features of host resistance linked to hilar fat deposition.

There are several potential mechanisms by which excess hilar fat may contribute to an increased likelihood of nodal metastases. Structurally, excess hilar adipose may compress traversing arteries, veins, and efferent lymphatics, potentially compromising nodal function by decreasing vascular flow and lymphatic clearance of isolated tumor cells. A similar mechanism of fat compression in obesity has been described in the kidney, an organ that is structurally very similar to lymph nodes, wherein excess renal sinus fat compression of vessels has been linked to renal dysfunction and hypertension [31]. Increased hilar fat may additionally support the establishment of axillary metastases via mechanisms related to changes in the LN adipose microenvironment as described in other ectopic fat depots. These mechanisms include chronic low grade inflammation and alterations in lipid metabolism within obese adipose tissue that support the establishment and proliferation of malignant tumors [7, 32–36].

Our study is limited to obese patients as a preliminary investigation into the potential association between nodal fat deposition and axillary metastases. Excess adiposity that exceeds accumulation within classic subcutaneous fat depots is deposited within ectopic locations in and around organs including increased visceral fat, muscle fat, and liver steatosis [37]. There are local pro-tumorigenic effects unique to ectopic fatty microenvironments of obesity including changes in adipokines and dysregulated fatty lipid metabolism that have been linked to cancer progression [37–39]. Recent studies show that fat deposition within lymph nodes is strongly associated with obesity as defined by BMI. However, BMI does not accurately reflect fat distribution, and future studies are needed to investigate if the observed association between fat-enlarged nodes and nodal metastasis is also identified in non-obese women with breast cancer.

Our study is limited by its retrospective nature. Mammograms and breast MRI were obtained in different imaging centers; however, patients were referred to a single academic institution for breast cancer treatment, which may limit generalizability. Due to the limited size of the patient population at our institution, all obese node-positive patients diagnosed in our study period were collected as cases. Positive axillary metastases are known to be associated with more advanced tumor size and grade, and this was confirmed in our dataset. We adjusted for these potential confounding tumor characteristics in a multivariate model, a common statistical technique used in many studies. We hope that with larger study populations in future studies, a matched case–control study can be conducted to confirm our results. Also, at our institution, the decision to obtain pre-treatment and pre-operative breast MRI varies according to tumor molecular subtype, known nodal metastases, baseline study prior to neoadjuvant systemic therapy, and surgeon preference; and these factors may have introduced bias. Despite these potential biases, the observed association between increased LN size in the contralateral axilla on breast MRI and nodal metastasis in obese women was also confirmed in the mammographic analysis. Furthermore, our study indicates strong agreement of LN measurements on breast MRI between two independent breast imagers. We plan to confirm our findings with larger, multi-institutional future studies through external collaborations to improve our understanding of mechanisms behind fatty LNs responsible for the poor prognosis of breast cancer in obese women. We also plan to investigate the association between fatty LN and long-term breast cancer prognosis, including survival and cancer recurrence, with a larger patient cohort and long-term follow-up in the future.

To summarize, we observed a strong positive association between fat-enlarged axillary LN and axillary metastasis in obese women with breast cancer. While fatty nodes represent a benign variant relative to metastatic nodes, our findings suggest that enlarged fat-expanded axillary LNs may represent an imaging biomarker of axillary metastases in obese women.

## Supplementary Information

Below is the link to the electronic supplementary material.

Supplementary file1 (DOC 276 kb)

Supplementary file2 (DOC 105 kb)

## Data Availability

The datasets generated during and/or analyzed during the current study are available from the corresponding author on reasonable request.

## Code availability

Statistical analysis of this study was conducted using R version 4.0.3 and the code is available from the corresponding author on reasonable request.
