# Supplementary material for: Fat-enlarged axillary lymph nodes are associated with node-positive breast cancer in obese patients
Source: Breast Cancer Res Treat. Author manuscript; Available in PMC 2021 Aug 1. (PMC8302552; doi:10.1007/s10549-021-06262-z)
Supplement: Supplementary Material1 [file NIHMS1721178-supplement-Supplementary_Material1.doc]

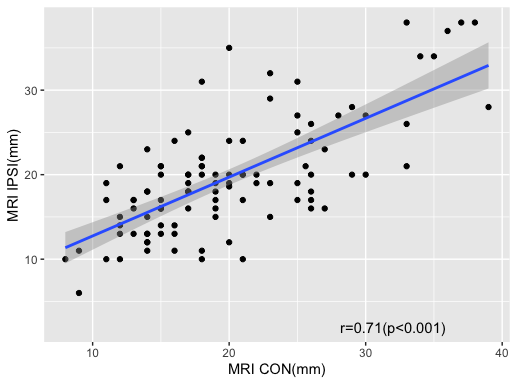


**Figure 1. Correlation between ipsilateral and contralateral LN.** LN measurement from contralateral vs ipsilateral axilla in node-negative patients demonstrated a strong positive correlation between node sizes in both axilla with a Pearson correlation coefficient of 0.71 (p<0.001). IPSI = ipsilateral, CON = contralateral.


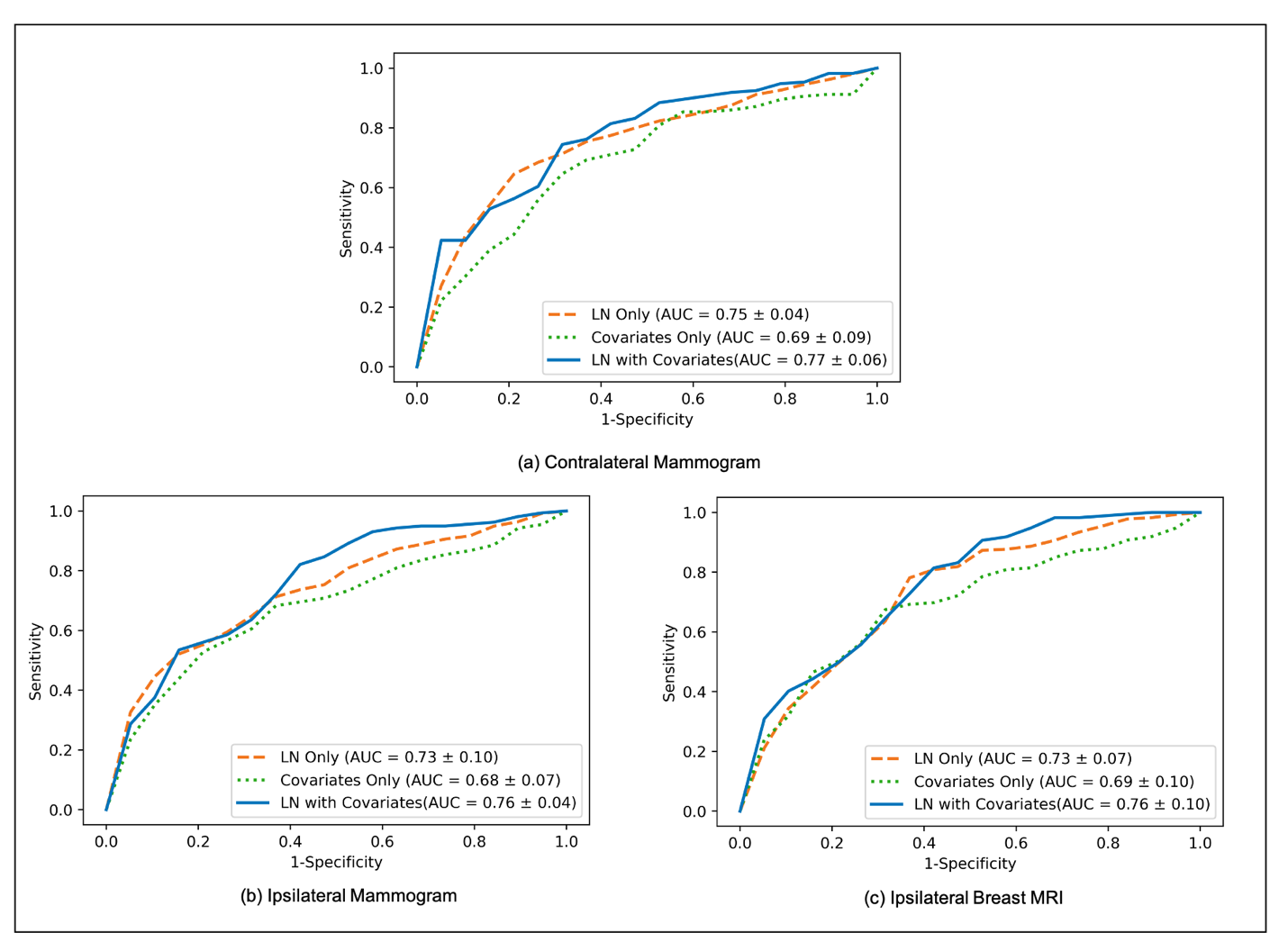


**Figure 2.** Mean ROC curves of node-positive breast cancer prediction with 5-fold cross-validation using LN size measured from (a) contralateral mammogram, (b) ipsilateral mammogram, and (c) ipsilateral breast MRI. Orange dashed lines indicate the predictions using contralateral LN size alone. Green dotted lines indicate the predictions using collected variables including patients’ age and BMI at diagnosis, tumor size, tumor grade, molecular subtype, and LVI. Blue solid lines indicate the predictions using LN size combined with other variables.
